# An analysis of spatial distribution characteristics and driving factors of traditional villages in the Loess Plateau

**DOI:** 10.1371/journal.pone.0329356

**Published:** 2025-08-01

**Authors:** Xiaobin Bao, Yingna Yang, Jianqing Qi, Yanbing He

**Affiliations:** 1 School of Architecture and Art Design, Henan Polytechnic University, Jiaozuo, Henan, China; 2 School of Marxism, Henan Polytechnic University, Jiaozuo, Henan, China; Wuhan University of Technology, CHINA

## Abstract

By revealing the spatial distribution characteristics and driving mechanisms of 1,027 traditional Chinese villages in the Loess Plateau, as announced in six batches up to 2023, this article provides a theoretical basis for formulating scientific and differentiated protection and development strategies. Utilizing the ArcGIS 10.8 platform and the GeoDetector model, this study comprehensively applies methods including the nearest neighbor index, standard deviation ellipse, kernel density estimation, and spatial autocorrelation to systematically analyze the spatial pattern of traditional villages, and quantitatively reveals their key driving factors and interactions through the GeoDetector. The results show: (1) Traditional villages in the Loess Plateau present a significant clustered spatial distribution (Nearest Neighbor Index R = 0.47, Moran’s I = 0.189), with an overall pattern of “dense in the east and west, sparse in the center,” primarily concentrated within an elliptical area with an flattening ratio of 0.664 and a directional angle of 84.5°, and with high-density areas located in Shanxi Province, the Yellow River basin along the Shanxi-Shaanxi border, and the Hehuang Valley. (2) Spatially, five major core clusters are formed, presenting a layered structure of “core clustering, gradient transition,” and exhibiting a non-stable diffusion trend towards the periphery. (3) The spatial differentiation is a result of the synergistic driving of human and natural factors, with human factors having a stronger overall explanatory power. Among them, the density of cultural heritage sites (q = 0.546) is the primary driving factor, followed by annual precipitation (q = 0.458), population size (q = 0.457), and urbanization rate (q = 0.354). The interaction between factors is significant and mostly shows non-linear enhancement, for instance, the interactive explanatory power of population size and the density of cultural heritage sites is as high as 0.852.

## 1 Introduction

Traditional villages, originally referred to as ancient villages, primarily denote settlements established prior to the Republic of China era that retain key elements such as traditional rural environments, architectural styles, and street layouts. These villages represent invaluable heritage and a profound testament to China’s agrarian civilization, which has evolved over millennia. Their preservation and development hold significant importance for the advancement of socialism with Chinese characteristics in the new era. However, due to factors such as accelerated urbanization, a considerable number of traditional villages are facing decline. Consequently, there is an urgent need to strengthen research and protection efforts for these villages. In recent years, China has placed considerable emphasis on the preservation and development of traditional villages. On April 16, 2012, the Ministry of Housing and Urban-Rural Development, along with three other departments, jointly issued the Notice on the Investigation of Traditional Villages, marking the official commencement of the investigation and identification of traditional villages in China. As of March 19, 2023, a total of 8,155 traditional villages have been officially recognized and listed across six batches of the National Catalogue of Traditional Villages. The Loess Plateau, one of the pivotal cradles of Chinese civilization, is home to 1,027 traditional villages, accounting for 12.6% of the total number of traditional villages in China. The region is noted for its unique loess landforms, long history of human habitation, and relatively fragile ecological environment. The traditional villages of the Loess Plateau are an ideal region for studying human-environment relationships and cultural adaptability, and their dense distribution in itself constitutes a phenomenon worthy of investigation.

Traditional villages constitute one of the primary subjects of study in disciplines such as human geography and architecture. As early as the first half of the 19th century, international scholars began to focus on rural settlements. For instance, Kohl explored the relationship between village locations and topographical features [1]; Meitzen investigated the formation mechanisms of rural settlements in northern Germany [2]; and Brunhes analyzed the interplay between villages and their surrounding environments [3]. Since the 20th century, research on rural settlements has predominantly revolved around themes such as settlement composition and development [4], as well as settlement morphology and classification [5], with qualitative analysis serving as the primary methodological approach [6]. However, the introduction of quantitative methods has significantly enhanced the precision of rural settlement studies [7,8]. Existing research has systematically reviewed the knowledge structure, mainstream topics, and emerging pathways of traditional villages between 1992 and 2025, revealing a significant growth trend in research output in this field. Studies show that China and EU member states have made outstanding contributions in this area, with more countries showing growing interest and influence. The international academic community generally agrees on the value and necessity of protecting traditional villages, with recent research focusing on cultural, historical, and ecological dimensions, and actively exploring sustainable local tourism development paths as a key trend for future development. The core themes of international research are mainly reflected in the following aspects: 1. Value and spatial cognition of traditional villages [9–13]: Focusing on the understanding of traditional villages as cultural landscapes, their multi-dimensional value assessment, and their environmental dependency and spatial characteristics. 2. Protection theory and practice [14,15]: Exploring the fundamental principles of heritage protection, the guiding significance of international standards and charters, as well as targeted conservation planning, restoration techniques, and management strategies. 3. Sustainable development and revitalization [16–19]: Focusing on the sustainable survival and development of traditional villages, especially promoting rural revitalization and community well-being through sustainable tourism, ecological agriculture, and cultural and creative industries, emphasizing the balance between development and protection. 4. Cultural change and community participation [20,21]: Studying the evolution and adaptation of the social structure, lifestyle, and cultural traditions of traditional villages under the impact of globalization and modernization, and emphasizing the participation mechanism and empowerment of the community as the main body of protection and development. 5. Application of digital technology [22]: Exploring the enabling role of digital technologies such as Geographic Information Systems (GIS), Remote Sensing (RS), Building Information Modeling (BIM), and Virtual Reality (VR) in heritage recording, monitoring, assessment, protection, display, and educational dissemination. Since the designation of traditional villages in China began in 2012, related research has been fruitful, with core themes mainly concentrated in the following areas: 1. Spatial distribution characteristics and influencing factors [23–26]: Research reveals the characteristics and influencing factors of the spatial distribution of traditional villages, with analyses covering both natural and human factors. The main purpose is to study what kind of environmental characteristics may be more conducive to the preservation of traditional villages; 2. Tourism development and revitalization [27–31]: Discussing the role and challenges of tourism in the revival of traditional villages, and introducing theoretical perspectives such as “landscape genes” to guide protection and development; 3. Human settlements and restoration transformation [32–41]: Focusing on the systemic composition and evolution of the human settlement environment in traditional villages, and exploring integrated restoration and development models; 4. Cultural heritage protection and landscape research [42–46]: Involving the synergistic protection of tangible and intangible cultural heritage, discussion of the “catalog” system, and comprehensive research treating traditional villages as cultural landscapes.

The limitations of existing research are mainly reflected in the lack of studies that conduct a systematic, multi-faceted, and quantitative analysis of the spatial distribution characteristics and their dual natural and human influencing factors for all six batches of traditional villages within the complete geographical unit of the Loess Plateau. Although national-level, large-scale studies have a broad perspective, they struggle to fully consider the unique human-environment relationship system of the Loess Plateau and its internal complexity. Current regional studies on the Loess Plateau are often geographically limited to specific municipalities, counties, river basins, or geomorphological units, such as Yulin in northern Shaanxi [47], the Yellow River basin along the Shanxi-Shaanxi border [48], and the Qingjian River basin [49]. These studies have not covered all six batches of the national traditional village catalog, leading to a lack of holistic regional understanding based on a complete dataset. This cognitive gap constrains the ability to formulate more scientific, targeted, and effective protection and sustainable development strategies for the traditional villages in this region. This knowledge gap is not merely a deficiency in data volume but, more critically, a lack of comprehensive and holistic understanding at the key regional scale. As a relatively complete geographical and cultural unit, the systematic study of traditional villages in the Loess Plateau needs to transcend administrative boundaries or isolated case studies, and the complete dataset used in this research, which includes all 1,027 villages covering all six batches of the catalog, is the key to moving from fragmented information to integrated regional cognition.

This study aims to systematically analyze the spatial distribution patterns of all six batches of 1,027 traditional villages in the Loess Plateau, and, by means of ArcGIS and GeoDetector, to quantitatively reveal the key natural and human driving factors influencing their spatial differentiation and their complex interaction mechanisms. Its core innovation lies in the first-time integration of data from all six batches of villages across the entire Loess Plateau and the in-depth analysis of factor interactions and intra-regional differences. On a theoretical level, this study is dedicated to deepening the theoretical understanding of human-environment relationships in specific historical-geographical regions, revealing how natural and human factors jointly shape the settlement patterns and evolution of ecologically fragile areas under a long history of settlement and significant environmental pressure, providing a detailed footnote for understanding long-term human settlement patterns in ecologically sensitive areas; promoting the application of cultural landscape ecology in areas with deep heritage, demonstrating how millennial human activities leave spatial imprints on a unique geographical unit, and explaining the spatial expression of cultural adaptation and persistence; and enriching the theoretical content of settlement sustainability research in ecologically fragile areas by quantifying factor interactions, constructing more realistic settlement evolution models, and providing new theoretical perspectives for the protection and sustainable development of settlements in vast, ecologically fragile areas with deep historical foundations.

## 2 Data sources and research methodology

### 2.1 Overview of the study area

**Fig 1:**
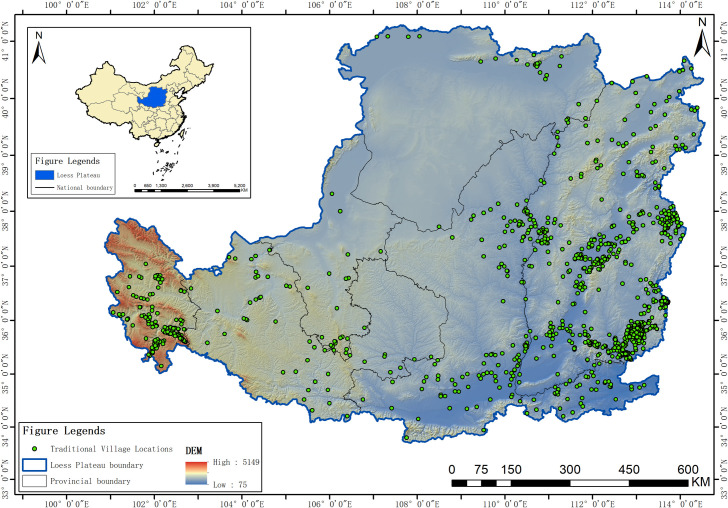
Distribution of traditional villages in the Loess Plateau region (Revision No. GS(2020)4619).

The Loess Plateau constitutes a relatively distinct geographical unit, encompassing the region west of the Taihang Mountains, east of the Riyue Mountains, north of the Qinling Mountains, and south of the Great Wall. Spanning approximately 1,300 kilometers from east to west and 800 kilometers from north to south, it covers a total area of about 635,000 square kilometers. This expansive region includes parts of seven provinces and autonomous regions: Qinghai, Gansu, Ningxia, Inner Mongolia, Shaanxi, Shanxi, and Henan [50]. The topography of the Loess Plateau slopes from the northwest to the southeast, characterized by a highly fragmented landscape with numerous ravines and gullies. Its climate is predominantly a continental monsoon type (Fig 1). The Loess Plateau was chosen as the study area due to its unique representativeness and scientific value; the region is noted for its vast territory, unique loess deposits, complex terrain of gullies and ravines, and severe ecological challenges. The traditional villages of the Loess Plateau are a vivid example of long-term human adaptation and the creation of civilization under immense environmental pressure; their settlement site selection, layout, architectural forms, and methods of resource utilization all profoundly reflect the interactive relationship between humans and the environment. Therefore, the Loess Plateau is not only representative due to its deep historical heritage and dense distribution of traditional villages, but more so because its highly challenging ecological background, which has shaped unique settlement patterns, provides an ideal “natural laboratory” for investigating the spatial characteristics of traditional settlements and the complex natural and human driving mechanisms behind them.

### 2.2 Data sources

The point location data for traditional villages were obtained using the Baidu Maps API coordinate picker, and their original coordinate system is BD-09. In this study, to ensure consistency with other geospatial data and the accuracy of subsequent spatial analysis, the acquired BD-09 coordinate data were uniformly converted to the internationally common WGS84 coordinate system. The converted WGS84 coordinates were used for all spatial analyses in this research. Although the precision of the original point locations provided by the Baidu Maps API may have certain variations, it is generally sufficient to meet the needs of this regional-scale study.

Digital Elevation Model (DEM) data with a resolution of 30 meters were sourced from the Geospatial Data Cloud (https://www.gscloud.cn/home). Vector boundary data for the Loess Plateau were acquired from the Digital Journal of Global Change Data Repository (https://www.geodoi.ac.cn/edoi.aspx?DOI=10.3974/geodb.2015.01.09.V1). The sources and timeliness of data for natural factors, such as climate and hydrology, as well as for social and cultural influencing factors, are shown in Table 1. The time span of these datasets is from 2018 to 2023. In addressing these temporal differences, this study selected the latest and most comprehensive data available for each factor at the time of analysis. It must be acknowledged that this time frame introduces a degree of temporal heterogeneity. For example, soil type data from 2018 is considered relatively stable over this short period, whereas socioeconomic indicators such as GDP, population size, and urbanization rate provide a relevant contemporary overview for the traditional villages listed up to 2023.

**Table 1:** Sources and timeliness of impact factors.

Type of impact factor	Impact factor	Indicator	Timeliness of data	Source of data
Natural factor	Topographic conditions	Topographic data	2023	Geospatial Data Cloud
		Soil type	2018	National Earth System Science Data Center
	Climatic conditions	Annual precipitation	2023	Zenodo
		Annual average temperature	2023	National Earth System Science Data Center
	Hydrological conditions	Annual average relative humidity	2023	National Earth System Science Data Center
		River density	2023	Earth Big Data Science Data Center
Humanistic factor	Demographic conditions	Population size	Main Data of the Seventh National Population Census	(China) National Bureau of Statistics
	Economic conditions	GDP	2023	(China) National Bureau of Statistics
		Urbanization rate	2023	(China) National Bureau of Statistics
		Nighttime Lighting Data	2023	Harvard Dataverse
	Transport condition	Road data	2023	National Geographic Center of China
	Historical and Cultural Conditions	Location of heritage conservation units	National key protected cultural relic units	National Cultural Heritage Administration

### 2.3 Research methodology

#### 2.3.1 Nearest Neighbor Analysis.

Nearest Neighbor Analysis (NNA) is a point pattern analysis method used to examine the spatial relationships among point locations [51]. The nearest neighbor distance quantifies the degree of proximity between point features within a geographic space. The Nearest Neighbor Index (NNI) is defined as the ratio of the observed average nearest neighbor distance to the expected average distance, which facilitates the identification of the primary spatial distribution characteristics and attributes of a point set. The formula is as follows:


$$\begin{array}{c} R=\frac{{\overset{―}{r}}_{i}}{{\overset{―}{r}}_{E}}=2\sqrt{D}\times {\overset{―}{r}}_{i} \end{array}$$ (1)

$$\begin{array}{c} {\overset{―}{r}}_{E}=\frac{1}{2\sqrt{\frac{m}{A}}}=\frac{1}{2\sqrt{D}} \end{array}$$ (2)

In the formula: R represents the Nearest Neighbor Index; $\;{\bar{r}}_{E}$ denotes the theoretical nearest neighbor distance; ${\bar{r}}_{i}$ signifies the observed nearest neighbor distance; m 为 indicates the number of point elements; A corresponds to the area of the region containing the point elements; D represents the point density. When R < 1, it suggests that the traditional villages exhibit a clustered distribution pattern. When R=1, it indicates that the traditional villages demonstrate a random distribution pattern. When R>, it implies that the traditional villages display a uniform distribution pattern.

#### 2.3.2 Geographic Concentration Index.

The Geographic Concentration Index (GCI) is one of the methodologies employed to analyze the spatial distribution patterns of point data, particularly suitable for evaluating the distribution of point-based elements, such as traditional villages, across different administrative regions. The formula is as follows:


$$\begin{array}{c} G=100\sqrt{\sum_{i=1}^{n}{\left(\frac{X_{i}}{T}\right)}^{2}} \end{array}$$ (3)

In the formula: G represents the Geographic Concentration Index; $X_{i}$ denotes the number of traditional villages in the i-th province (or autonomous region) within the Loess Plateau; T signifies the total number of traditional villages; n corresponds to the total number of provinces (or autonomous regions). The value of G ranges between [0, 100], where a higher G indicates a more concentrated distribution, while a lower G suggests a more dispersed distribution. Let $G_{0}$ represent the Geographic Concentration Index under the assumption that traditional villages are uniformly distributed across all provinces (or autonomous regions). If $G\;>\;G_{0}$, the distribution is considered concentrated; otherwise, it is relatively dispersed.

#### 2.3.3 Imbalance Index.

The Imbalance Index serves as a quantitative measure to assess the degree of distributional equilibrium of traditional villages across various provinces (or autonomous regions). The formula is as follows:


$$\begin{array}{c} S=\frac{\sum_{i=1}^{n}Y_{i}-50(n+1)}{100\times n-50(n+1)} \end{array}$$ (4)

In the formula: S represents the Imbalance Index; n denotes the number of provinces (or autonomous regions); $Y_{i}$ signifies the cumulative percentage of traditional villages in the i-th province (or autonomous region) after ranking the proportions of traditional villages from largest to smallest. The value of S ranges between [0, 1]. When =0, it indicates that traditional villages are uniformly distributed across all regions. As S approaches 0, the spatial distribution of traditional villages becomes more dispersed. As S approaches 1, the distribution becomes more concentrated. When S=1, it implies that traditional villages are entirely concentrated within a single province (or autonomous region).

#### 2.3.4 Standard Deviation Ellipse.

The Standard Deviation Ellipse (SDE) method is employed to quantitatively elucidate the global and spatial characteristics of the distribution of traditional villages in the Loess Plateau region, encompassing aspects such as centrality, dispersion, orientation, and spatial morphology. The formula is as follows:


$$\begin{array}{c} X_{SDE}=\sqrt{\frac{\sum_{i=1}^{n}{\left(x_{i}-\overset{―}{x}\right)}^{2}}{n}} \end{array}$$ (5)

$$\begin{array}{c} Y_{SDE}=\sqrt{\frac{\sum_{i=1}^{n}{\left(y_{i}-\overset{―}{y}\right)}^{2}}{n}} \end{array}$$ (6)

$$\begin{array}{c} \tan\theta =\frac{\sum_{i=1}^{n}{\tilde{x}}_{i}^{2}\sum_{i=1}^{n}{\tilde{y}}_{i}^{2}+\sqrt{\left(\sum_{i=1}^{n}{\tilde{x}}_{i}^{2}-\sum_{i=1}^{n}{\tilde{y}}_{i}^{2}\right)}+{\left(4\sum_{i=1}^{n}{\tilde{x}}_{i}^{2}{\tilde{y}}_{i}^{2}\right)}^{2}}{2\sum_{i=1}^{n}{\tilde{x}}_{i}{\tilde{y}}_{i}} \end{array}$$ (7)

$$\sigma_{x}=\sqrt{\frac{\left(\sum {\tilde{x}}_{i}^{2}+\sum {\tilde{y}}_{i}^{2}\right)+\sqrt{{\left(\sum {\tilde{x}}_{i}^{2}-\sum {\tilde{y}}_{i}^{2}\right)}^{2}+4{\left(\sum {\tilde{x}}_{i}{\tilde{y}}_{i}\right)}^{2}}}{2n}}$$ (8)

$$\sigma_{y}=\sqrt{\frac{\left(\sum {\tilde{x}}_{i}^{2}+\sum {\tilde{y}}_{i}^{2}\right)-\sqrt{{\left(\sum {\tilde{x}}_{i}^{2}-\sum {\tilde{y}}_{i}^{2}\right)}^{2}+4{\left(\sum {\tilde{x}}_{i}{\tilde{y}}_{i}\right)}^{2}}}{2n}}$$ (9)

$$F=1-\frac{\sigma_{y}}{\sigma_{x}}$$ (10)

$$e=\sqrt{1-\frac{(\sigma_{y}{)}^{2}}{(\sigma_{x}{)}^{2}}}$$ (11)

In the formula: $x_{i}$ and $y_{i}$ are the coordinate values of the i-th element; $\overset{―}{x}$ and $\overset{―}{y}$ represent the mean center of the elements; n is the total number of elements. The closer the flattening ratio F is to 1, the more flattened the ellipse; the closer it is to 0, the closer it is to a circle. The value of eccentricity e is between 0 and 1, and a larger value indicates a more flattened ellipse.

#### 2.3.5 Kernel Density Estimation.

The Kernel Density Estimation (KDE) method is utilized to assess the aggregation patterns of traditional villages across the entire Loess Plateau region. The formula is as follows:


$$\begin{array}{c} f(x)=\frac{1}{nh}\sum_{i=1}^{n}k\left(\frac{x-x_{\dot{l}}}{h}\right) \end{array}$$ (12)

In the formula: f(x) represents the kernel density value; $k\left(\frac{x-x_{\dot{l}}}{h}\right)$ denotes the kernel function; h signifies the bandwidth; n corresponds to the number of point elements; $x-x_{\dot{l}}$ indicates the distance from the estimation point x to the event $x_{\dot{l}}$. In this study, the Kernel Density Estimation method was used in the ArcGIS 10.8 platform to analyze the spatial clustering patterns of traditional villages. The analysis employed the software’s default Quartic kernel function. The determination of the key parameter, bandwidth (h, also known as search radius), adopted an adaptive method based on the spatial distribution characteristics of the data (Silverman’s Rule of Thumb), which was automatically calculated by the software according to the distribution and number of the input point features.

#### 2.3.6 Spatial autocorrelation analysis.

(1) Global Spatial Autocorrelation

This study uses the Global Moran’s I to measure the overall trend of the spatial distribution of traditional villages throughout the Loess Plateau region. Its calculation formula is:


$$I=\frac{n\sum_{i=1}^{n}\sum_{j=1}^{n}w_{ij}(x_{i}-\bar{x})(x_{j}-\bar{x})}{\left(\sum_{i=1}^{n}\sum_{j=1}^{n}w_{ij}\right)\sum_{i=1}^{n}(x_{i}-\bar{x}{)}^{2}}$$ (13)

In the formula, n is the total number of traditional villages; $x_{i}$ and $x_{j}$ are the attribute values for village i and j, respectively (in this study, these are the kernel density values reflecting the degree of clustering or the grid-based count of villages); $\bar{x}$ is the average of all village attribute values; and $w_{ij}$ is the spatial weight matrix. The value range for the Moran’s I index is [−1, 1]. An index greater than 0 indicates positive spatial correlation, meaning the traditional villages have a clustered distribution; less than 0 indicates negative spatial correlation, representing a dispersed distribution; and a value close to 0 indicates a random distribution. The statistical significance of the result is typically determined in conjunction with the Z-score and p-value.

(2) Getis-Ord Gi*

To precisely identify the specific spatial locations of high-value clusters (hot spots) and low-value clusters (cold spots), this study further employs Hot Spot Analysis (Getis-Ord Gi*). This method identifies whether a statistically significant spatial clustering of attribute values exists for each feature with its neighboring features by calculating the Gi* statistic for each feature. Its calculation formula is:


$$G_{i}^{*}=\frac{\sum_{j=1}^{n}w_{ij}x_{j}-\bar{X}\sum_{j=1}^{n}w_{ij}}{S\sqrt{\frac{\left[n\sum_{j=1}^{n}w_{ij}^{2}-(\sum_{j=1}^{n}w_{ij}{)}^{2}\right]}{n-1}}}$$ (14)

In the formula, $x_{j}$ is the attribute value of feature j, $w_{ij}$ is the spatial weight between feature i and j, n is the total number of features, and $\bar{X}$ and S are the mean and standard deviation of the attribute values, respectively. For the $G_{i}^{*}$ statistic, a higher Z-score indicates a tighter clustering of high values (hot spots); conversely, a lower Z-score indicates a tighter clustering of low values (cold spots).

#### 2.3.7 GeoDetector.

The GeoDetector is a statistical tool designed to identify spatial heterogeneity within geographic elements and to uncover their driving factors. Its core principle posits that if an independent variable significantly influences a dependent variable, the spatial distributions of the two variables should exhibit a high degree of similarity [52]. The formula is as follows:


$$\begin{array}{c} q=1-\frac{\sum_{h=1}^{L}n_{h}\sigma_{h}^{2}}{n\sigma^{2}} \end{array}$$ (15)

In the formula: L represents the stratification of the dependent variable Y or the independent variable X, defined as classification or partitioning; $n_{h}$ and $\sigma_{h}^{2}$ denote the number of units and the variance within stratum h, respectively; n and σ correspond to the total number of units and the overall variance in the study area; The q−valueranges between [0, 1], where a higher value indicates stronger explanatory power of the independent variable on the dependent variable. In this study, natural and socio-cultural factors influencing the distribution of traditional villages were extracted, and an indicator system was constructed across three dimensions: environmental, economic, and cultural. The study uses the GeoDetector for Microsoft Excel 2018 software tool to conduct factor detection and interaction detection analysis. When interpreting the detection results, this study adopts a significance level threshold of p < 0.05. When a factor’s p-value is less than 0.05, that factor has statistically significant explanatory power for the spatial differentiation of traditional villages. Additionally, to test for potential multicollinearity issues among the driving factors and thus ensure the reliability of the model results, this study conducted a collinearity diagnosis on the independent variables using the Variance Inflation Factor (VIF) and Tolerance before performing the GeoDetector analysis.

## 3 Characteristics of spatial differentiation of traditional villages in the Loess Plateau region

### 3.1 Characterization of spatial distribution types

**Fig 2:**
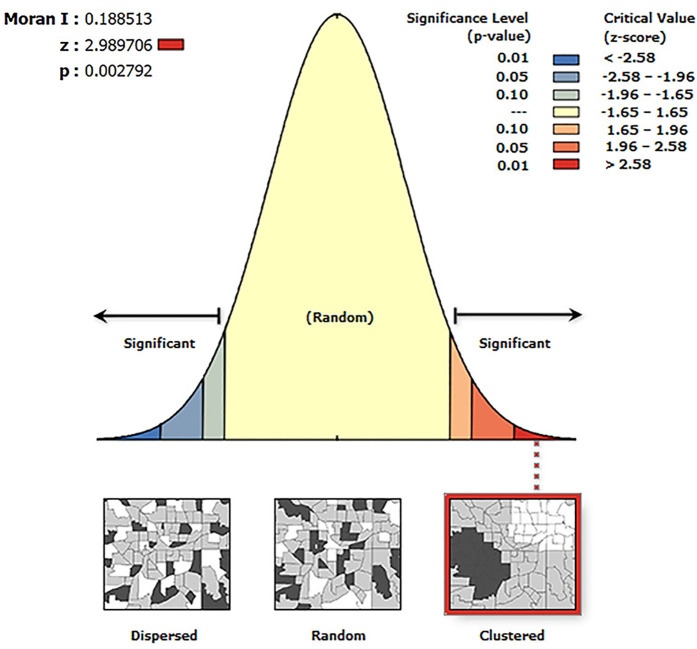
Spatial autocorrelation statement of traditional villages in the Loess Plateau region.

**Fig 3:**
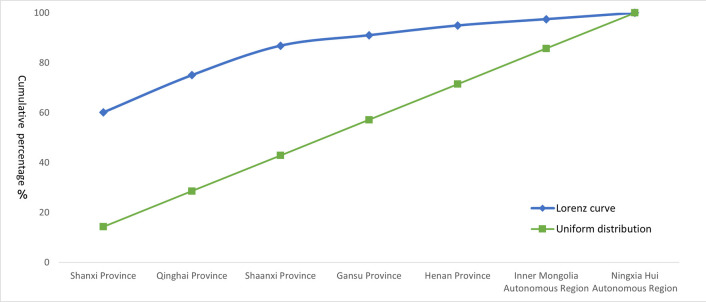
Lorenz Curve of Spatial Distribution of Traditional Villages in Loess Plateau Region.

**Fig 4:**
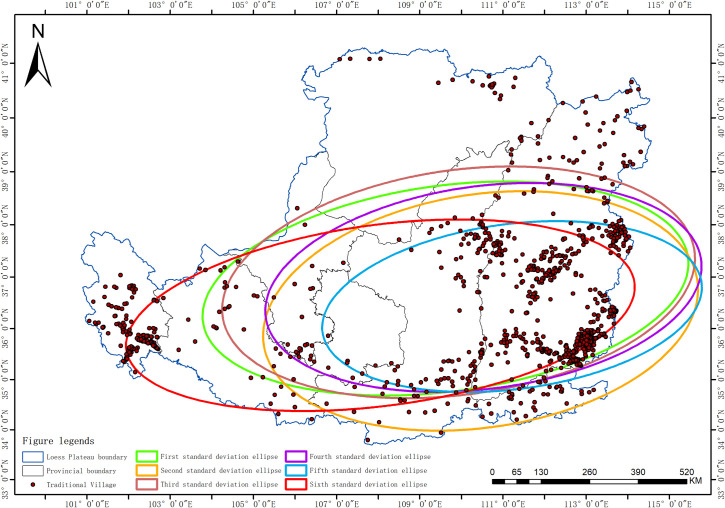
Ellipse analysis plot of standard deviation of traditional villages in the Loess Plateau region (Revision No. GS(2020)4619).

The Average Nearest Neighbor analysis shows a Z-score of −32.66, a P-value of 0.00, and R = 0.47 < 1, indicating that the spatial distribution of traditional villages in the Loess Plateau region is of a clustered type. To further statistically validate this clustering characteristic, this study introduced global spatial autocorrelation analysis. According to the spatial autocorrelation report (Fig 2), the Moran’s I for the distribution of traditional villages in the Loess Plateau is 0.189, which is a significant positive value. At the same time, the Z-score is 2.990, and the p-value is 0.003. At a 0.01 significance level, the null hypothesis that the spatial distribution of traditional villages is random can be rejected. The Z-score is much greater than the critical value of 2.58, indicating that the traditional villages in the Loess Plateau show a very significant state of clustering in space. The total number of traditional villages T=1027, and the number of provinces (or autonomous regions) n=7. The Geographic Concentration Index G=63.46, while the expected index under uniform distribution $G_{0}=37.80$. The result $G>G_{0}$ confirms that the distribution of traditional villages is highly concentrated, particularly in Shanxi Province, which accounts for 60.18% of the total (Table 2). The Imbalance Index S=0.685 further underscores the uneven spatial distribution of traditional villages, characterized by a “dense east-west, sparse central” pattern. Specifically, Shanxi, Qinghai, and Shaanxi provinces constitute the primary concentration areas, collectively accounting for over 80% of the total traditional villages within the study region (Fig 3). The standard deviation ellipse of traditional village distribution exhibits a low flattening ratio, with a distinct aggregation effect and directional orientation. The villages are predominantly concentrated within an elliptical region with a flattening ratio of 0.664 and a directional angle of 84.5°, displaying an approximate “east-west” alignment (Fig 4). This “dense east-west, sparse central” clustered distribution pattern exerts a dual influence on regional socioeconomic development: Spatial agglomeration stimulates the establishment of cultural tourism corridors, enhances resource integration and industrial symbiosis, improves infrastructure cost-efficiency, and fosters scale economies, consequently accelerating specialized industrial cluster development. While generating these synergistic effects, the pattern concurrently exacerbates regional development discrepancies, elevates overdevelopment risks in high-density zones, and induces asymmetric resource allocation patterns.

**Table 2:** Number of Traditional Villages in Each Province of the Loess Plateau Region.

Province Name	Number of Traditional Villages	Percentage
Shanxi Province	618	60.18%
Qinghai Province	153	14.90%
Shaanxi Province	121	11.78%
Gansu Province	43	4.19%
Henan Province	40	3.89%
Inner Mongolia Autonomous Region	26	2.53%
Ningxia Hui Autonomous Region	26	2.53%
Total	1027	100.00%

### 3.2 Hot spot detection of spatial distribution

**Fig 5:**
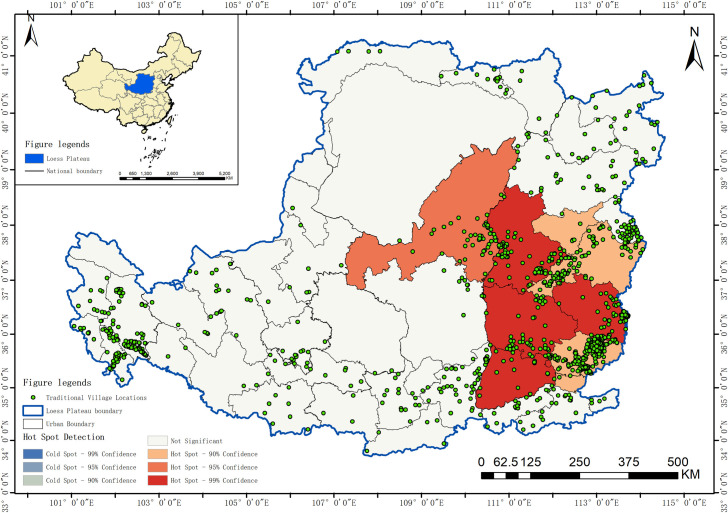
Hotspot analysis of traditional villages in the Loess Plateau region (Revision No. GS(2020)4619).

The preceding text confirmed that traditional villages have a significant clustering trend. To further precisely identify the specific spatial locations of these clusters and their statistical significance, this study conducted a Hot Spot Analysis (Getis-Ord Gi*), the results of which are shown in Fig 5.

The analysis results clearly reveal the extreme imbalance of the spatial distribution of traditional villages in the Loess Plateau, presenting a “single core - large periphery” clustering pattern. In the Fig, a vast and statistically extremely significant core hot spot area is formed only in the east. This area highly coincides with the geographical outline of Shanxi Province, and its core zone (mainly central-southern Shanxi) reaches a 99% confidence level, surrounded by secondary hot spot zones with 95% and 90% confidence, displaying a clear layered structure. In contrast, for the vast central, western, and northern regions, including the Hehuang Valley in Qinghai, their village distributions are all statistically “not significant” areas, failing to form any statistically meaningful clustering centers. Meanwhile, no significant cold spot areas were detected within the study area, which further reinforces the spatial differentiation characteristic of traditional villages being composed of a “super core” and a vast “randomly sparse zone”.

### 3.3 Spatial distribution density characteristics

**Fig 6:**
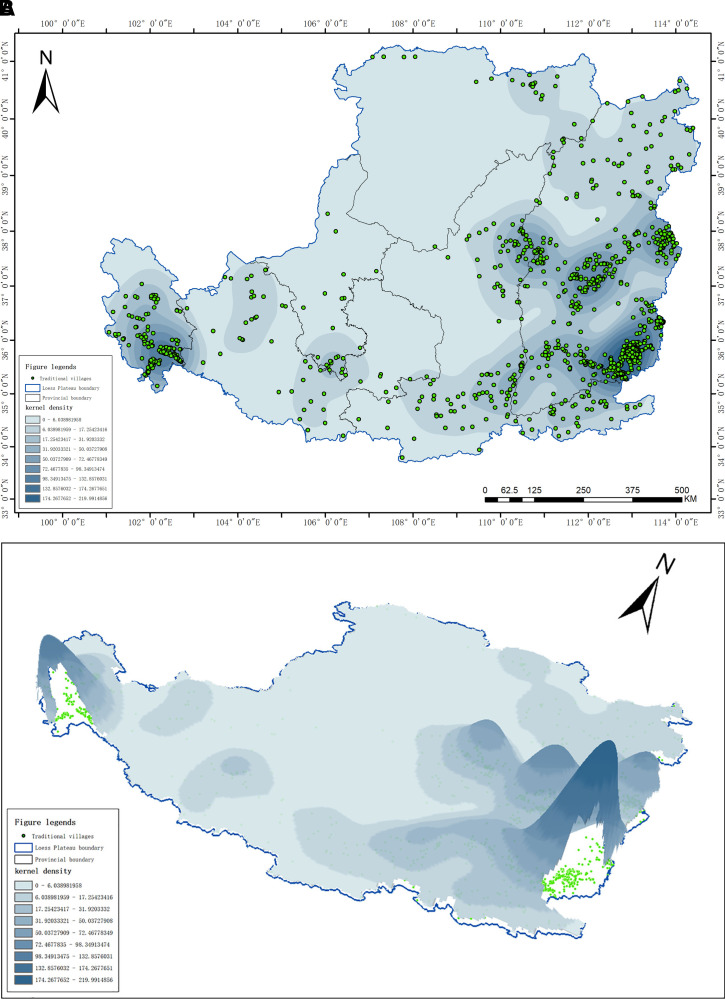
Kernel density map of traditional villages in the Loess Plateau region (Revision No. GS(2020)4619). a. Traditional village kernel density analysis b. 3D visualization and analysis of kernel density in traditional villages map.

The spatial distribution of traditional villages in the Loess Plateau region exhibits a pronounced clustered pattern, characterized by a “core aggregation with gradient transition” spatial structure. Five distinct core clusters are prominently observed, predominantly located along the periphery of the study area (Fig 6). Notably, the core regions are situated in Shanxi Province, the Yellow River basin along the Shanxi-Shaanxi border, and the Hehuang Valley. These areas are closely associated with factors such as policy support, economic development levels, transportation accessibility, and historical heritage. Shanxi Province, benefiting from robust policy support and strong conservation awareness, has implemented over 100 policy documents following the launch of national initiatives to strengthen the protection of traditional villages, thereby establishing a solid foundation for their preservation and forming the highest density zone [53]. The Hehuang Valley, due to its relatively remote location and limited socio-economic development, has preserved a significant degree of authenticity in its villages, largely undisturbed by external influences. Meanwhile, the Yellow River basin along the Shanxi-Shaanxi border boasts a rich historical and cultural heritage, resulting in a high concentration of traditional settlements.

## 4 Drivers of spatial differentiation in traditional villages

### 4.1 Model specification and pre-testing

#### 4.1.1 Construction of influencing factor system and collinearity diagnosis.

The formation of the spatial pattern of traditional villages is comprehensively influenced by multiple factors such as topography, hydrology, transportation, and economy. Referencing past studies, this research extracts 15 potential driving factors influencing the distribution of traditional villages from two aspects, natural conditions and human conditions, to construct an indicator system from three dimensions: environmental, economic, and cultural (Table 3) [48,54]. The natural environment is the material basis for village site selection, determining the basic conditions for survival and development; human factors, especially economic development and cultural inheritance, profoundly affect the evolution, revitalization, and spatial clustering of villages.

**Table 3:** Indicator System for Factors Influencing the Spatial Differentiation of Traditional Villages.

Type of Impact Factor	Impact Factor	Indicator	Indicator Interpretation
Natural factor	Topographic conditions	X1/ Elevation	Elevation affects the climate and accessibility of villages
		X2/ Aspect	Aspect affects insolation and microclimatic conditions
		X3/ Gradient	Gradient affects land use and agricultural production in villages
		X4/ Soil type	Soil type affects local agricultural development
	Climatic conditions	X5/ Annual precipitation	Precipitation affects agricultural production and water availability
		X6/ Annual average temperature	Temperature affects agricultural cultivation and residential comfort
	Hydrological conditions	X7/ Annual average relative humidity	Humidity affects human comfort and agricultural activity
		X8/ River density	River density affects village accessibility and irrigation conditions
Humanistic factor	Demographic conditions	X9/ Regional population size	Population size affects the number of villages
	Economic conditions	X10/ Regional GDP	GDP reflects the level of regional economic development
		X11/ Urbanization rate of the region	The urbanization rate reflects the ratio of urban to rural areas
		X12/ Light intensity at night	Reflecting human economic activity and settlement intensity
	Transport condition	X13/ Distance to highway	Highways affect accessibility
		X14/ Distance from general road	General highways affect accessibility
	Historical and Cultural Conditions	X15/ Density of cultural heritage preservation sites	Richness of the historical and cultural heritage of the region

The multicollinearity diagnosis results show that for the 7 human driving factors included in this study, all Variance Inflation Factor (VIF) values are far less than 5, and all Tolerance values are greater than 0.2. The results indicate that no serious multicollinearity problem exists among the driving factors. Based on this diagnosis, we decided to retain the three variables of population, GDP, and urbanization rate in the main model to comprehensively examine the independent explanatory power of each factor.

#### 4.1.2 Influencing factor sensitivity analysis.

This study takes the results of the kernel density analysis of traditional villages as the dependent variable representing their spatial differentiation pattern. The influencing factors, as independent variables, need to be discretized to meet the input requirements of the GeoDetector model. The study uses the natural breaks method to uniformly classify each driving factor into 9 categories. Considering that the choice of the number of classes may be subjective and could affect the model results, to ensure the robustness and reliability of this study’s conclusions, we conducted a systematic sensitivity analysis of this method. By comprehensively comparing the changes in the classification breaks and the GeoDetector q-values for the influencing factors under scenarios with <9 classes, 9 classes, and >9 classes (Table 4), the core conclusion drawn is: the scenario with more than 9 classes led to greater result fluctuations, while the scenario with fewer than 9 classes might weaken the explanatory power of some factors. Therefore, the 9-class scenario, as a more balanced, conservative, and risk-tested intermediate option, is the most appropriate methodological choice for this study.

**Table 4:** Summary of Multi-scenario Sensitivity Analysis for Core Driving Factors.

Influencing Factor	Breakpoint Sensitivity Assessment	q-value (9)	q-value (>9)	q-value Change Rate (>9)	q-value (<9)	q-value Change Rate(<9)	Overall Assessment
X15/Density of cultural heritage sites	Relatively robust	0.546	0.567	3.85%	0.584	7.01%	Relatively robust
X5/Annual precipitation	Robust	0.458	0.232	−49.37%	0.512	11.80%	Relatively sensitive
X9/Regional population	Relatively robust	0.457	0.441	−3.42%	0.564	23.41%	Relatively robust
X11/Urbanization rate	Robust	0.354	0.358	1.18%	0.435	22.81%	Relatively robust
X6/Annual average temperature	Relatively robust	0.287	0.291	1.32%	0.295	2.78%	Robust
X10/Total GDP	Highly sensitive	0.228	0.007	−96.91%	0.033	−85.63%	Results unreliable
X4/Soil type	Robust	0.214	0.216	0.55%	0.216	0.55%	Robust
X8/River density	Robust	0.185	0.182	−1.39%	0.193	4.69%	Robust
X1/Elevation	Robust	0.143	0.147	2.29%	0.165	15.14%	Relatively sensitive
X7/Annual relative humidity	Relatively robust	0.094	0.081	−14.77%	0.089	−6.21%	Relatively sensitive
X13/Distance to highway	Robust	0.091	0.095	3.92%	0.096	5.48%	Robust
X12/Nighttime light intensity	Robust	0.079	0.078	−0.69%	0.082	3.29%	Robust
X14/Distance to general road	Robust	0.051	0.056	9.11%	0.068	33.22%	Relatively sensitive
X3/Slope	Robust	0.03	0.028	−7.12%	0.029	−3.09%	Robust
X2/Aspect	–	0.006	0.007	21.75%	0.008	44.69%	Consistently not significant

### 4.2 Single-factor detection

The results of the single-factor driving analysis (Table 5) show that among the 15 potential driving factors, except for X2/Aspect (p = 0.826) which is not statistically significant, the p-values for the remaining 14 potential factors are all less than 0.001, indicating that these factors have a significant impact on the spatial differentiation of traditional villages. Among these, 9 driving factors have a q-value greater than 0.1, indicating high explanatory power. Ranked by explanatory power (q-value), they are: X15/Density of cultural heritage sites (0.546) > X5/Annual precipitation (0.458) > X9/Regional population size (0.457) > X11/Regional urbanization rate (0.354) > X6/Annual average temperature (0.287) > X10/Regional GDP (0.228) > X4/Soil type (0.214) > X8/River density (0.185) > X1/Elevation (0.143). Among these factors, X15/Density of cultural heritage sites, X5/Annual precipitation, X9/Regional population size, and X11/Regional urbanization rate are the core factors with the strongest explanatory power for the spatial differentiation of traditional villages. Areas with a high density of cultural heritage sites (X15) have accumulated richer cultural heritage during their historical process, thus having the strongest explanatory power. As a key natural element, the influence of X5/Annual precipitation is second only to the density of cultural heritage sites, and it has an important impact on the formation and development of villages by affecting agricultural production and the living environment. Overall, the comprehensive explanatory power of human factors is higher than that of natural factors, which may be related to the fact that the differences in human development among the provinces in the study area are more significant than the differences in the natural environment.

**Table 5:** Results of the Factor Detector for the Influence (q-value) of Various Impact Factors.

Indicator	q-value	p-value
X1/ Elevation	0.143	p < 0.001
X2/ Aspect	0.006	p = 0.826
X3/ Gradient	0.03	p < 0.001
X4/ Soil type	0.214	p < 0.001
X5/ Annual precipitation	0.458	p < 0.001
X6/ Annual average temperature	0.287	p < 0.001
X7/ Annual average relative humidity	0.094	p < 0.001
X8/ River density	0.185	p < 0.001
X9/ Regional population size	0.457	p < 0.001
X10/ Regional GDP	0.228	p < 0.001
X11/ Urbanization rate of the region	0.354	p < 0.001
X12/ Light intensity at night	0.079	p < 0.001
X13/ Distance to highway	0.091	p < 0.001
X14/ Distance from general road	0.051	p < 0.001
X15/ Density of cultural heritage preservation sites	0.546	p < 0.001

### 4.3 Multi-factor coupling analysis

Multi-factor coupling analysis aims to reveal the interactions between different driving factors. In this analysis, “non-linear enhancement” is a key concept, and its criterion is: the interaction effect q-value produced when two factors act together is greater than the sum of the q-values of their independent effects, i.e., q(X1∩X2)>q(X1)+q(X2). The analysis results show that the two-factor interactions between factors generally exhibit non-linear enhancement characteristics, and their coupled explanatory power is significantly higher than that of any single factor (Table 6).

**Table 6:** Results of the Factor Detector for the Coupled Influence (q-value) of Multiple Factors.

因子	X1	X2	X3	X4	X5	X6	X7	X8	X9	X10	X11	X12	X13	X14	X15
X1	0.143														
X2	0.203	0.006													
X3	0.235	0.082	0.030												
X4	0.369	0.243	0.257	0.214											
X5	0.606	0.479	0.495	0.646	0.458										
X6	0.436	0.340	0.369	0.482	0.630	0.287									
X7	0.300	0.131	0.177	0.366	0.619	0.422	0.094								
X8	0.439	0.237	0.273	0.381	0.587	0.512	0.464	0.185							
X9	0.545	0.495	0.513	0.652	0.716	0.618	0.584	0.683	0.457						
X10	0.432	0.302	0.294	0.472	0.734	0.550	0.382	0.527	0.576	0.228					
X11	0.525	0.383	0.421	0.604	0.727	0.665	0.455	0.649	0.678	0.614	0.354				
X12	0.437	0.159	0.175	0.395	0.667	0.591	0.281	0.401	0.634	0.469	0.655	0.079			
X13	0.340	0.147	0.166	0.340	0.566	0.450	0.232	0.420	0.616	0.344	0.547	0.310	0.091		
X14	0.218	0.096	0.132	0.273	0.498	0.335	0.194	0.283	0.484	0.308	0.408	0.182	0.219	0.051	
X15	0.685	0.591	0.626	0.713	0.780	0.700	0.625	0.673	0.852	0.701	0.802	0.741	0.619	0.619	0.546

Note: Red shading represents non-linear enhancement of the two-factor interaction, blue shading represents linear enhancement of the two-factor interaction.

**Fig 7:**
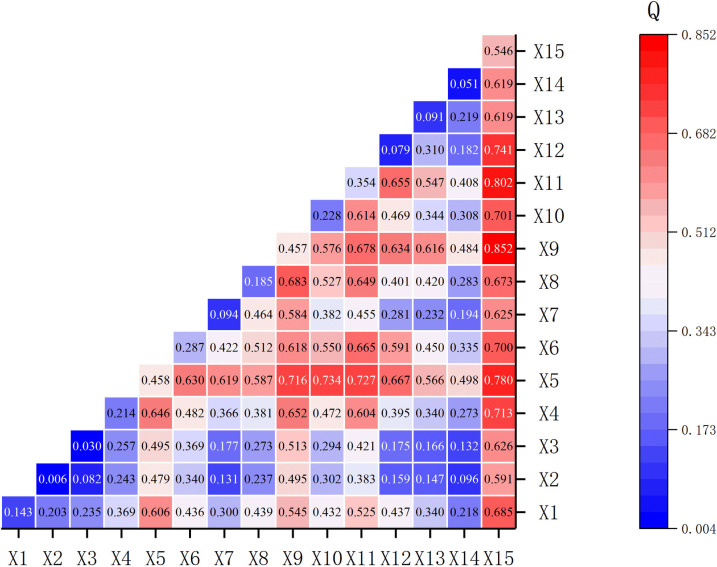
Multi-factor coupling effect results (q-value) heat map.

Specifically, the most significant strong coupling combinations include: X9/Population size and X15/Density of cultural heritage sites (q = 0.852), X11/Urbanization rate and X15/Density of cultural heritage sites (q = 0.802), and X5/Precipitation and X15/Density of cultural heritage sites (q = 0.780). At the same time, combinations such as X5/Precipitation and X6/Temperature (q = 0.630), and X4/Soil type and X11/Urbanization rate (q = 0.604) form moderately strong couplings (Fig 7). In contrast, the coupling effects between purely natural elements, such as X2/Aspect and X1/Elevation (q = 0.203), are relatively weak. This complex coupling relationship reflects the synergistic driving mechanism of human and natural elements on the spatial pattern of traditional villages. For example, precipitation directly affects agricultural production and population carrying capacity, while the density of cultural heritage sites and the level of regional economic development mutually reinforce each other, forming a positive feedback loop. These factors are interwoven into a complex driving network that shapes the distribution characteristics of traditional villages. Therefore, when formulating protection strategies, this multi-factor coupling effect must be considered, and differentiated measures tailored to local conditions must be adopted.

### 4.4 Analysis of the influence mechanism of the dominant factor

#### 4.4.1 Density of cultural heritage preservation sites.

**Fig 8:**
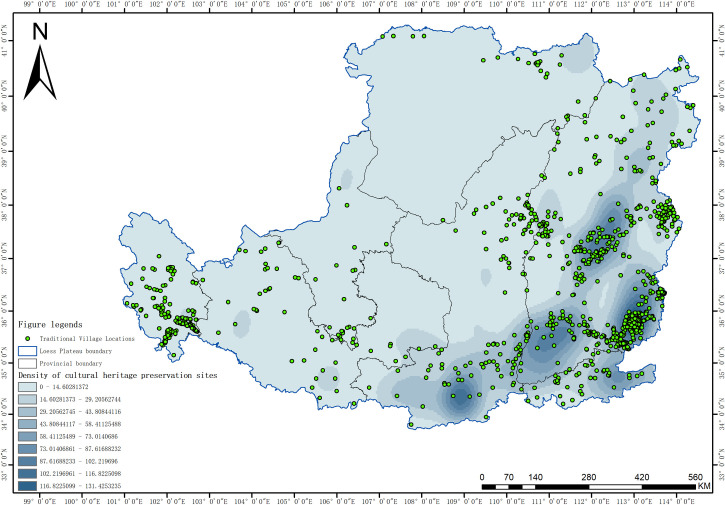
Relationship between spatial distribution of traditional villages and density of cultural heritage preservation sites in the Loess Plateau region (Revision No. GS(2020)4619).

The density of cultural heritage preservation sites, identified as the primary driving factor (q = 0.546) for the spatial heterogeneity of traditional villages in the Loess Plateau region, reflects the concentration of regional historical and cultural heritage. It reveals the intrinsic relationship between the distribution of traditional villages and the spatial patterns of historical and cultural resources (Fig 8). Spatially, the density of cultural heritage preservation sites and the distribution of traditional villages exhibit significant spatial coupling characteristics. Both form a triangular high-value zone in the central-southeastern Shanxi and northern Shaanxi regions, representing a core area with profound historical and cultural accumulation. Additionally, a belt-shaped distribution along the Yellow River in Shanxi and Shaanxi, as well as along major transportation corridors, highlights the historical influence of river valley civilizations and key trade routes. The mechanisms underlying this spatial correlation can be interpreted from the following perspectives: regions with a high density of cultural heritage preservation sites possess a rich historical foundation, providing the cultural bedrock for the formation and continuity of traditional villages. Furthermore, the spatial agglomeration effect between cultural heritage sites and traditional villages creates a positive feedback loop, collectively shaping an integral component of the regional cultural landscape. This “culture-space” composite mechanism underscores the density of cultural heritage preservation sites as a critical indicator for understanding the spatial heterogeneity of traditional villages in the Loess Plateau. In the practice of traditional village conservation, it is essential to fully consider the spatial distribution characteristics of cultural heritage preservation sites. A regional cultural protection network should be established, with these sites serving as pivotal anchors. Differentiated protection strategies should be tailored to local conditions: in high-density areas, the focus should be on strengthening the overall protection framework; in transitional zones, emphasis should be placed on constructing cultural corridors; and in low-density areas, efforts should prioritize cultivating the unique characteristics of individual villages. This approach will facilitate the synergistic conservation and sustainable development of traditional villages and regional cultural heritage.

#### 4.4.2 Annual precipitation.

**Fig 9:**
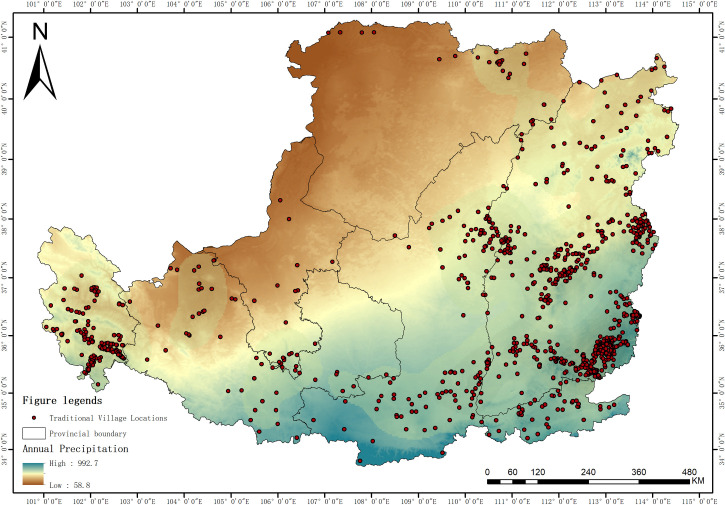
Relationship between spatial distribution of traditional villages and Annual Precipitation in Loess Plateau region (Revision No. GS(2020)4619).

Annual precipitation, as a key natural influencing factor in the spatial differentiation of traditional villages (q = 0.458), exhibits a distinct gradient characteristic, decreasing from southeast to northwest (Fig 9). The overall range of precipitation values is between 58.8 and 992.7 mm. The macro-distribution of traditional villages and the precipitation isohyets show a high degree of spatial coupling, mainly manifesting as a differentiation feature with three gradient levels: In areas with abundant annual precipitation (>600mm), mainly concentrated in the Guanzhong Plain and southeastern Shanxi in the southeastern corner of the study area, the most core dense distribution belt of traditional villages is formed. The superior hydrothermal conditions in these areas are the cornerstone for the prosperity of traditional farming civilization. In areas with moderate annual precipitation (400–600 mm), covering most of central Shanxi, northern Shaanxi, and eastern Gansu, traditional villages are mostly distributed in clusters or along river valleys. And in areas with scarce annual precipitation (<400mm), i.e., the vast northwestern region, the harsh arid environment greatly restricts human settlement activities, and traditional villages only show a scattered, point-like distribution. Annual precipitation profoundly shapes the spatial pattern of traditional villages by directly determining the region’s agricultural production potential and ecological carrying capacity. Abundant precipitation is a prerequisite for developing dryland farming, ensuring food security, and maintaining a large population size in the Loess Plateau region, providing the most fundamental material basis for the “birth-survival-development” of traditional villages. Therefore, precipitation isohyets, especially the 400 mm isohyet, have historically often served as the dividing line between farming and nomadic civilizations, and naturally became the “lifeline” determining whether traditional farming settlements could be densely distributed. Under this decisive influence, traditional villages in different precipitation zones show significant differences in scale, density, and even livelihood patterns.

#### 4.4.3 Population size.

**Fig 10:**
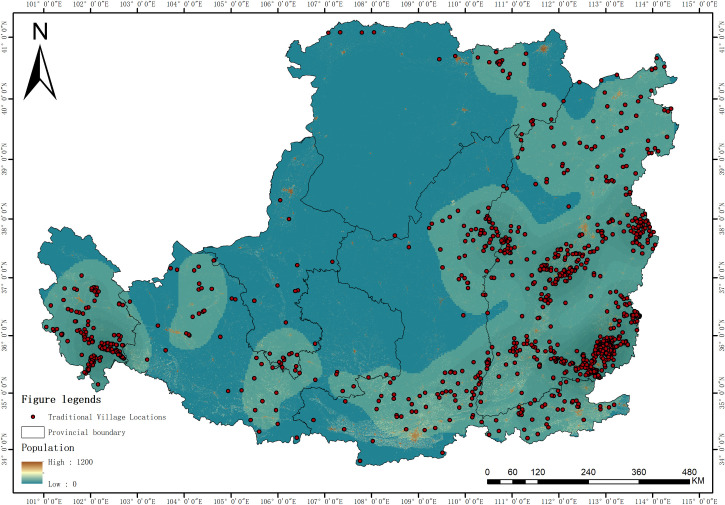
Relationship between spatial distribution of traditional villages and Population Size in the Loess Plateau (Revision No. GS(2020)4619).

Population size, as a key driving factor in the spatial differentiation of traditional villages in the Loess Plateau region (q = 0.457), exhibits a significant positive correlation (Fig 10). Traditional villages are highly clustered in areas with larger populations, such as the Guanzhong Plain and the Jinzhong Basin. As one of the important cradles of Chinese civilization, the Loess Plateau, especially the Guanzhong region centered on Xi’an, was the political and cultural center for 13 dynasties including Zhou, Qin, Han, Sui, and Tang, which formed a stable foundation for population aggregation. Favorable natural conditions (such as plains and river valleys) provided good environmental carrying capacity for population accumulation and village development. From the perspective of interaction mechanisms, population size affects the spatial distribution of traditional villages through three dimensions: first, the population scale effect, where a larger population base provides the necessary social foundation for the survival of traditional villages; second, the cultural inheritance effect, where densely populated areas often have stronger cultural inertia and continuity of tradition; third, the economic support effect, where higher population density is usually accompanied by stronger economic capacity, providing material support for the protection and renewal of traditional villages. Therefore, in the practice of traditional village protection, different strategies should be adopted according to local conditions: for densely populated areas, the focus should be on the living conservation and functional enhancement of traditional villages; for areas with moderate population density, efforts should be focused on cultivating distinctive villages and consolidating their development foundations; for sparsely populated areas, the emphasis should be on protecting the basic features and preventing the trend of hollowing out. At the same time, a management mechanism that combines population change monitoring with the dynamic protection of traditional villages should be established to achieve the sustainable development of traditional villages.

#### 4.4.4 Urbanization rate.

**Fig 11:**
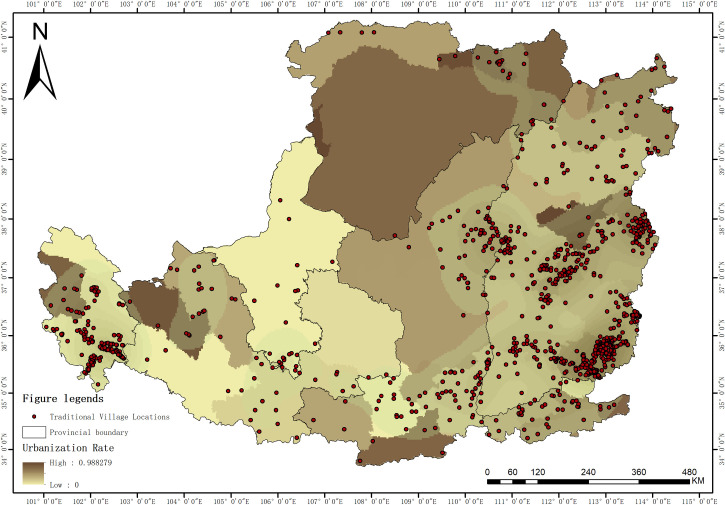
Relationship between spatial distribution and Urbanization Rate of traditional villages in the Loess Plateau region (Revision No. GS(2020)4619).

Urbanization rate, as an important driving factor in the spatial differentiation of traditional villages in the Loess Plateau region (q = 0.354), exhibits a significant “inverted U-shaped” characteristic (Fig 11). Traditional villages are mainly concentrated in moderately urbanized areas with an urbanization rate of 40%−60%, forming two typical regions represented by the Guanzhong city cluster and the Taiyuan city cluster. From the perspective of its mechanism, the driving effect of urbanization on the spatial distribution of traditional villages is bidirectional: on the one hand, an excessively high urbanization rate (>70%) may lead to an exacerbated outflow of the rural population and severe rural hollowing, as seen in the relatively low density of traditional villages in the areas surrounding Xi’an; on the other hand, a low level of urbanization (<40%), due to a weak economic foundation and insufficient conservation awareness, struggles to support an effective protection system for traditional villages, as is the case in some remote areas of Qinghai. Moderately urbanized areas often have a more balanced urban-rural development pattern, capable of providing necessary economic support and demographic security for traditional villages. These areas have usually established relatively complete cultural heritage protection systems, including special funding support, technical guidance, and management frameworks. At the same time, the coupling effect brought by the positive interaction of urban and rural elements promotes the organic integration of traditional villages with modern development, such as the prosperous development of rural tourism. In the practice of traditional village protection, a “region-specific policy” strategy should be adopted: for highly urbanized areas, the focus should be on preventing the encroachment of urban expansion on traditional villages and strengthening spatial governance; for moderately urbanized areas, efforts should be on improving the protection system and promoting the two-way flow of urban and rural elements; for areas with low urbanization, the priority should be to actively cultivate protection capacity and establish basic safeguarding mechanisms. At the same time, a development model that coordinates the graded advancement of urbanization with the protection of traditional villages should be established to achieve a positive interaction between the preservation of traditional culture and the urbanization process.

## 5 Discussion

### 5.1 Model robustness test

To address concerns about the theoretical correlation among socioeconomic indicators such as population, GDP, and urbanization rate within the GeoDetector model, we conducted a robustness test by constructing simplified models. The specific procedure is as follows: we took the model containing all driving factors as the full model and constructed three additional simplified models with different settings for comparison. Simplified Model A retains only “urbanization rate” as the representative indicator of socioeconomic development, removing population and GDP; Simplified Model B retains only “population size,” removing urbanization rate and GDP; while Simplified Model C retains only “GDP,” removing urbanization rate and population. The comparison results (Table 7) show that in all four models (i.e., one full model and three simplified models), “density of cultural heritage sites” is consistently the driving factor with the strongest explanatory power, and its q-value remains at a high level in all cases. At the same time, the relative importance ranking of other key natural and human factors, such as annual relative humidity and annual average temperature, also maintained a high degree of consistency. The socioeconomic factors themselves, whether appearing individually or simultaneously, also showed stable q-values and significance.

**Table 7:** Comparison of Robustness Check Results under Different Model Specifications.

Influencing Factor	Full Model q-value	Simplified Model A (Urbanization Rate Only) q-value	Simplified Model B (Population Only) q-value	Simplified Model C (GDP Only) q-value
X15/Density of cultural heritage sites	**0.546**	**0.546**	**0.546**	**0.546**
X5/Annual precipitation	**0.458**	**0.458**	**0.458**	**0.458**
X9/Provincial population	**0.457**	—	**0.457**	—
X11/Urbanization rate	**0.354**	**0.354**	—	—
X6/Annual average temperature	**0.287**	0.287	0.287	0.287
X10/Total GDP	**0.228**	—	—	**0.228**
X4/Soil type	0.214	0.214	0.214	0.214
X8/River density	0.185	0.185	0.185	0.185
X1/Elevation	0.143	0.143	0.143	0.143
X7/Annual relative humidity	0.094	0.094	0.094	0.094
X13/Distance to highway	0.091	0.091	0.091	0.091
X12/Nighttime light intensity	0.079	0.079	0.079	0.079
X14/Distance to general road	0.051	0.051	0.051	0.051
X3/Slope	0.03	0.03	0.03	0.03
X2/Aspect	0.006	0.006	0.006	0.006

This series of diagnoses and tests demonstrates the robustness and reliability of this study’s conclusions. The relative importance of the driving factors found in the study, especially the core conclusion of “density of cultural heritage sites” as the primary driving force, is objective and credible, and is not caused by specific model specifications or collinearity between variables.

### 5.2 Theoretical elucidation of factor-driving mechanisms

The core finding of this study is that the spatial differentiation of traditional villages is driven by both human and natural factors, but human factors, especially cultural factors, play a dominant role. This conclusion is not just a simple presentation of data; its deeper reasons can be explained through the theoretical frameworks of cultural geography and evolutionary economic geography. From the perspective of cultural geography, the dominant position of “X15/Density of cultural heritage sites” as the factor with the strongest explanatory power (q = 0.546) is not accidental. A traditional village is itself a “cultural landscape” that carries collective memory and local identity. As one of the important cradles of Chinese civilization, the Loess Plateau has deep cultural accumulation. Areas with a high density of cultural heritage sites signify the continuity and strength of cultural inheritance, forming a powerful “cultural path dependence.” Under this mechanism, local societies have a stronger will and internal drive to protect traditional settlements that carry their historical information, thus enabling these villages to be preserved amidst the wave of modernization. Therefore, the distribution pattern of villages is a spatial projection of millennial-scale cultural selection and consolidation. From the perspective of evolutionary economic geography, the strong coupling effects among multiple factors reveal a “co-evolution” mechanism of regional development. For example, the extremely strong coupling of “X9/Population size” and “X15/Density of cultural heritage sites” (q = 0.852), as well as the coupling of “X11/Urbanization rate” and “X15/Density of cultural heritage sites” (q = 0.802), reflects a typical “lock-in effect.” Historically, areas that were culturally prosperous and suitable for habitation attracted population aggregation (high population and high density of cultural heritage), and this aggregation, in turn, spurred early urbanization, thereby locking in a path of synergistic development between culture and socioeconomic society. Natural elements play a foundational and constraining role in this process. For example, the strong coupling of “X5/Annual precipitation” with multiple human factors indicates that in the Loess Plateau, a region with relatively scarce water resources, the evolutionary path of human society has always been rigidly constrained by hydrological conditions.

At the same time, comparing this study with related research in Southwest China [55] can reveal fundamental regional differences in the driving mechanisms of traditional villages. The most core difference lies in the type of dominant factor: in the Southwest, due to its extremely fragmented terrain, the distribution of villages is dominated by natural environmental factors such as elevation and rivers; whereas in the Loess Plateau, an important origin of Chinese civilization, its spatial pattern is dominated by the cultural-historical factor of “density of cultural heritage sites,” reflecting a deep cultural path dependence. This difference in dominant factor type is also reflected in factor interactions; the strongest coupling in the Southwest occurs between natural elements, whereas in the Loess Plateau, the coupling of human and cultural elements is the strongest. Although studies in both regions show a commonality of traditional villages being concentrated in economically underdeveloped areas, the core new insight derived from the comparison is: the dominant driving force for the spatial distribution of traditional villages is not universally constant, but rather undergoes a fundamental shift from “nature-dominated” to “culture-dominated” based on differences in the depth of regional geography and history.

### 5.3 Differentiated development strategies for traditional village clusters

The five core clusters identified in this study show significant differences in terms of natural environmental endowments, historical and cultural foundations, and regional economic locations. This profound diversity dictates that we must not adopt a simple, uniform, “one-size-fits-all” protection model. On the contrary, it is necessary to deeply analyze the intrinsic characteristics and development constraints of each cluster, and based on their unique regional conditions and resource endowments, tailor precise and differentiated protection and development strategies. Only in this way can we ensure that protection measures are truly implemented and take root, effectively stimulate the endogenous vitality of each cluster, and achieve the sustainable protection of cultural heritage or ecological resources and the synergistic progress of regional development.

#### 5.3.1 Jinzhong-Taiyuan City Cluster Periphery Cluster.

This cluster is adjacent to the provincial capital city, giving it a prominent locational advantage. Its development strategy should focus on the integration of cultural tourism, wellness, and research study tours in the metropolitan periphery. The development direction is to leverage its convenient transportation and vast market advantages, focusing on developing high-value-added business formats for the city’s middle- and high-income groups, such as weekend getaways, wellness and retirement living, research study tours for primary and secondary school students, and creative agriculture, while avoiding the construction of low-level, homogenized farm-stay businesses. For budget and funding sources, it is recommended to actively connect with the policy loans and subsidies of the National Urban-Rural Integration Development Pilot Zones, and to establish special funds to attract social capital for market-oriented investment through an “enterprise + village collective” model. The responsible agencies should be led by the municipal-level Culture and Tourism Bureaus and the Planning and Natural Resources Bureaus of both Taiyuan and Jinzhong cities, which will formulate a strict list of permitted industrial implants and guidelines for architectural style control. Performance indicators can be set as: an average annual increase of 10% in the non-agricultural income share of villagers within the cluster over the next three years; an average tourist stay of more than 1.5 days; and the proportion of market-operated cultural tourism projects among the total projects.

#### 5.3.2 Southeast Shanxi - Northwest Henan Border Cluster.

This cluster spans both Shanxi and Henan provinces, with magnificent ancient fortress architectural complexes and a profound Jin Merchant culture as its core features. Its development strategy should be positioned on the revitalization of ancient architecture and the revival of Jin Merchant culture. The development direction is to take leading scenic spots like the Royal Residence of the Prime Minister as the core, linking surrounding ancient villages to form a “grand fortress courtyard + idyllic ancient village” collaborative development model, with a focus on promoting the transformation of idle Jin Merchant residences into new business formats such as boutique B&Bs, artist studios, and contract and document museums. Budget and funding should aim to secure special funds for the protection and utilization of provincial ancient architecture and promote public-private partnership (PPP) models, such as “right to repair in exchange for right to operate,” to attract private capital. For the responsible agency, it is recommended to establish a “Shanxi-Henan Ancient Village Collaborative Development Working Group” with the participation of relevant municipal governments such as Jincheng in Shanxi and Jiaozuo in Henan, responsible for joint marketing and project coordination. Performance indicators should include: the number of successfully revitalized Ming and Qing dynasty ancient courtyards in the region; the annual occupancy rate of high-end boutique B&Bs; and the annual number of tourists diverted from the core scenic spots to surrounding villages.

#### 5.3.3 East Shanxi - Hebei Border Cluster.

This cluster is located at the eastern foot of the Taihang Mountains and is a strategic corridor connecting the North China Plain and the Loess Plateau, possessing a unique pass and border culture. Its development strategy should focus on the integration of the Taihang Mountains’ natural scenery with the Great Wall pass culture. The development direction is to integrate regional historical resources such as Niangzi Pass and the Guguang Great Wall, combined with the natural scenery of the Taihang Mountains, to develop tourism products themed on ancient trail hiking, pass nostalgia, and military history experiences, and to proactively integrate into the Beijing-Tianjin-Hebei tourism market. Budget and funding should actively align with the relevant plans for the Great Wall National Cultural Park and the Taihang Mountains tourism industry development to secure construction funds at the national and provincial levels. The responsible agencies should be led by the relevant municipal governments of Yangquan in Shanxi and Shijiazhuang in Hebei, especially the culture, tourism, and sports departments, to jointly develop cross-regional hiking routes and annual tourist passes. Performance indicators can be set as: the annual growth rate of tourists from the Beijing-Tianjin-Hebei region; the amount of media coverage for cultural brands such as the “Taihang Eight Trails”; and the added value of the sports tourism and cultural tourism industries within the region.

#### 5.3.4 Shanxi-Shaanxi Yellow River Bank Cluster.

The lifeline of this cluster is the Yellow River, and its culture and landscape are both closely linked to it. Its development strategy should be centered around the “Yellow River Story” national cultural brand. The development direction is to deeply explore the cultural elements of the villages along the bank, such as ferry crossings, boat trackers, water conservancy, and cave dwellings, to create an immersive Yellow River cultural experience belt that integrates the natural wonders of the Yellow River, farming civilization experiences, and red revolutionary holy sites. Budget and funding should be fully aligned with the national strategy for ecological protection and high-quality development of the Yellow River basin to secure ecological compensation and project construction funds from the central government. The responsible agencies should be jointly led by the provincial-level Yellow River basin management agencies and the Culture and Tourism Departments of both Shanxi and Shaanxi provinces, to coordinate ecological protection, waterway management, and cultural tourism development along the river. Performance indicators should include: the vegetation coverage rate and water quality compliance rate of the ecological corridor along the Yellow River; the number of Yellow River-related folk customs successfully declared as national intangible cultural heritage; and the annual self-driving tourist traffic on the tourism highway along the Yellow River.

#### 5.3.5 Qinghai Hehuang Valley Cluster.

This cluster is located on the northeastern edge of the Qinghai-Tibet Plateau, with a fragile ecological environment, and the integration of multiple ethnic cultures is its most valuable resource. Its development strategy must adhere to an ecology-first principle and the transmission of cultural authenticity. The development direction is to strictly control development intensity and to develop appointment-based, small-scale, high-value research study tourism centered on intangible cultural heritage learning, religious art exploration, and plateau ecological science popularization. The budget and funding should mainly rely on national fiscal transfers for ecological function zones such as Sanjiangyuan and the Qilian Mountains, and special funds for ethnic minority development, and it can also try to cooperate with internationally renowned ecological and cultural conservation foundations. The responsible agency must be a high-level joint approval and supervision committee composed of the provincial Department of Ecology and Environment, the Department of Culture and Tourism, and the Ethnic and Religious Affairs Commission, implementing a one-vote veto system for all projects. The performance indicators should not be the number of tourists, but rather: the activity level and number of apprentices of representative inheritors of “intangible cultural heritage”; the assessment results of the annual Gross Ecosystem Product (GEP); and the average consumption per tourist and their contribution to local culture.

### 5.4 Research limitations and prospects

Although this study has conducted a systematic analysis of the spatial differentiation and driving mechanisms of traditional villages in the Loess Plateau, some inherent limitations still exist. First, at the data level, the years of the driving factor data used in this study are not completely uniform (2018–2023), and this temporal heterogeneity may not fully capture the dynamic relationship between certain rapidly changing factors and the village patterns. At the same time, using county-level administrative units as the statistical unit for socioeconomic data may also introduce the Modifiable Areal Unit Problem (MAUP), and the scale sensitivity of the results requires further examination. Second, in terms of model methodology, although the GeoDetector model can effectively identify spatial associations and interactions, it is essentially a correlation-based analysis and struggles to fully reveal strict causal relationships. Potential endogeneity issues in the research, such as the possible bidirectional causality between economic development and village protection, cannot be handled by this model. Finally, this study uses the official catalog as its sample source, which is itself a purposeful selection process, and the generalizability of its conclusions to the vast number of unlisted traditional villages needs further validation.

Based on the above limitations, future research can be deepened and expanded in the following aspects. At the methodological level, long-term panel datasets can be constructed and dynamic models such as Geographically and Temporally Weighted Regression (GTWR) can be used to reveal the evolutionary process of village patterns; higher-resolution gridded socioeconomic data can be introduced to circumvent the potential impact of MAUP; and attempts can be made to use Structural Equation Modeling (SEM) or Multiscale Geographically Weighted Regression (MGWR) to conduct a more in-depth test of the causal mechanisms of the associations found in this study. At the content level, the research perspective can shift from macro-level pattern analysis to micro-level case studies, exploring the mechanisms of micro-factors such as clan structure, elite Figs, and local policy implementation through in-depth field surveys of typical villages. At the same time, research in historical geography should be strengthened, combining historical documents and archaeological findings to reconstruct the centennial or even millennial evolutionary history of village communities in key areas, thereby achieving a theoretical deepening from “pattern” to “process” and a more comprehensive understanding of the complex history of human-environment interaction in the unique geographical unit of the Loess Plateau.

## 6 Conclusion

(1) The spatial distribution pattern shows significant clustering and directionality. Spatial analysis finds that the 1,027 traditional villages in the Loess Plateau are not randomly scattered but exhibit a significant clustered form. This assertion is supported by multiple quantitative indicators: the Nearest Neighbor Index R value is 0.47 (<1), and the Global Spatial Autocorrelation Moran’s I index is 0.189 (p = 0.003), indicating that the clustering is extremely statistically significant. The overall distribution of the villages is concentrated within an elliptical area with a directional angle of 84.5° and an flattening ratio of 0.664, visually presenting a macro-pattern of being “dense in the east and west, sparse in the center.”
(2) The spatial clustering pattern manifests as a “multi-core, gradient” layered structure. On the basis of the aforementioned overall clustered pattern, kernel density analysis further reveals that the traditional villages are not uniformly clustered but have formed five major core density areas. These core areas and their peripheries constitute a spatial hierarchy of “core clustering, gradient transition,” and from there exhibit a non-stable diffusion pattern towards the surroundings, clearly reflecting the internal spatial structure of the village distribution.
(3) The spatial differentiation is synergistically driven by human and natural factors, with human factors playing a dominant role. The core driving factors with the strongest explanatory power are: Density of cultural heritage sites (q = 0.546), Annual precipitation (q = 0.458), Population size (q = 0.457), and Urbanization rate (q = 0.354). This strongly proves that deep historical and cultural accumulation is the primary condition for the survival of traditional villages, while a natural and social environment suitable for human settlement and farming is the foundation for their development and clustering. Furthermore, the interaction between factors can significantly enhance explanatory power, mostly manifesting as non-linear enhancement. For example, the explanatory power of the interaction between “Population size and Density of cultural heritage sites” is as high as 0.852. This finding reveals that the distribution of traditional villages is not the result of any single factor, but rather a spatial projection of the co-evolution and mutual reinforcement of culture, population, economy, and the natural background within a complex system.
